# COVID-19: Factors associated with symptoms of anxiety and depression in the ICU

**DOI:** 10.15649/cuidarte.3998

**Published:** 2025-03-26

**Authors:** Laura Andrea Díaz-Mayorga, Harol Giovanni Vivas-López, Claudia Consuelo Torres Contreras, Lyda Z. Rojas, Norma C. Serrano, Angie Yarlady Serrano-García, Margarita Rosa Parra Ortiz, Doris Cristina Quintero-Lesmes

**Affiliations:** 1 Universidad de Santander. Facultad de Ciencias Médicas y de la Salud. Instituto de Investigación Masira, Bucaramanga, Santander, Colombia. lauraandima2021@gmail.com Universidad de Santander. Facultad de Ciencias Médicas y de la Salud. Universidad de Santander Bucaramanga, Santander Colombia lauraandima2021@gmail.com; 2 Universidad de Santander. Facultad de Ciencias Médicas y de la Salud. Instituto de Investigación Masira, Bucaramanga, Santander, Colombia. haroldgiovanni06@gmail.com Universidad de Santander. Facultad de Ciencias Médicas y de la Salud. Universidad de Santander Bucaramanga, Santander Colombia haroldgiovanni06@gmail.com; 3 Universidad de Santander. Facultad de Ciencias Médicas y de la Salud. Instituto de Investigación Masira, Bucaramanga, Santander, Colombia. clau.torres@mail.udes.edu.co Universidad de Santander. Facultad de Ciencias Médicas y de la Salud. Universidad de Santander Bucaramanga, Santander Colombia clau.torres@mail.udes.edu.co; 4 Fundación Cardiovascular de Colombia, Floridablanca, Santander, Colombia. lydarojas@fcv.org Fundación Cardiovascular de Colombia Fundación Cardiovascular de Colombia Floridablanca, Santander Colombia lydarojas@fcv.org; 5 Fundación Cardiovascular de Colombia, Floridablanca, Santander, Colombia. normaserrano@fcv.org Fundación Cardiovascular de Colombia Fundación Cardiovascular de Colombia Floridablanca, Santander Colombia normaserrano@fcv.org; 6 Fundación Cardiovascular de Colombia, Floridablanca, Santander, Colombia. asgarcia96@gmail.com . angieserranogarcia@fcv.org Fundación Cardiovascular de Colombia Fundación Cardiovascular de Colombia Floridablanca, Santander Colombia asgarcia96@gmail.com; 7 Universidad Militar Nueva Granada, Bogotá DC, Colombia. est.margarita.parra@unimilitar.edu.co Universidad Militar Nueva Granada Universidad Militar Nueva Granada Bogotá DC Colombia est.margarita.parra@unimilitar.edu.co; 8 Fundación Cardiovascular de Colombia, Floridablanca, Santander, Colombia. dorisquintero@fcv.org Fundación Cardiovascular de Colombia Fundación Cardiovascular de Colombia Floridablanca, Santander Colombia dorisquintero@fcv.org

**Keywords:** Anxiety, Depression, Healthcare Personnel, Intensive Care Units, COVID-19, Ansiedad, Depresión, Personal de Salud, Unidades de Cuidados Intensivos, COVID-19, Ansiedade, Depressão, Pessoal de Saúde, Unidades de Terapia Intensiva, COVID-19

## Abstract

**Introduction:**

The COVID-19 pandemic led to a high prevalence of anxiety and depression among healthcare personnel.

**Objective:**

To assess the prevalence and independent risk factors associated with anxiety and depression symptoms among healthcare staff working in Intensive Care Units (ICUs) during the COVID-19 pandemic in Bucaramanga and its metropolitan area.

**Materials and Methods:**

This was an analytical cross-sectional study. Anxiety and depression were measured using the Hopkins Symptom Checklist-25 (HSCL-25). Bivariate and multivariate analyses were conducted using linear regressions to investigate associated factors.

**Results:**

A total of 288 people were included in the study. The prevalence of anxiety and depression symptoms was 8.34% (95% CI: 5.41-12.14%). In the bivariate analysis, six factors were associated with depression and anxiety symptoms; however, only three remained in the multivariate analysis: female sex (β=0.085, 95% CI: 0.019 - 0.151), experiencing COVID-19 symptoms in the past 14 days (β= 0.115, 95% CI: 0.024 - 0.205), and having worked in general ICUs and COVID-19 ICUs (β =0.009, 95% CI: 0.025 - 0.173).

**Discussion:**

The prevalence of anxiety and depression symptoms was considerably lower than reported in the scientific literature.

**Conclusion:**

In the studied population, although the prevalence of depression and anxiety symptoms was low, three independent factors were found to be statistically associated with the presence of these mental symptoms.

## Resumen

**Introducción:**

La pandemia por COVID-19 provocó una elevada prevalencia de ansiedad y depresión en el personal de la salud.

**Objetivo:**

Evaluar la prevalencia y los factores de riesgo asociados con los síntomas de ansiedad y depresión en personal de salud que trabaja en las unidades de cuidado intensivo (UCI) durante la pandemia de COVID-19 en Bucaramanga y su área metropolitana.

**Materiales y métodos:**

Se realizó un estudio transversal analítico. La ansiedad y la depresión se midieron usando la Hopkins Symptom Checklist-25 (HSCL-25). Se realizaron análisis bivariados y multivariados con regresiones lineales para identificar los factores asociados.

**Resultados:**

Se incluyó a un total de 288 personas. La prevalencia de síntomas de ansiedad y depresión fue de 8.34% (IC 95% 5.41-12.14%). En el análisis bivariado, seis factores fueron asociados con los síntomas de depresión y ansiedad, sin embargo, solo tres de ellos se mantuvieron en el análisis multivariado: ser mujer (β=0.085, IC 95% 0.019 - 0.151), síntomas de COVID-19 en los últimos 14 días (β= 0.115, IC 95% 0.024 - 0.205) y haber laborado en UCI general y UCI-COVID-19 (β =0.009, IC 95% 0.025 - 0.173).

**Discusión:**

La prevalencia de síntomas de ansiedad y depresión fue considerablemente menor a la reportada en la literatura científica.

**Conclusiones:**

En la población estudiada, aunque la prevalencia de síntomas de depresión y ansiedad fue baja, se encontraron tres factores independientes asociados estadísticamente con la presencia de estos síntomas mentales.

## Resumo

**Introdução:**

A pandemia de COVID-19 levou a uma alta prevalência de ansiedade e depressão entre os profissionais de saúde.

**Objetivo:**

Avaliar a prevalência e os fatores de risco independentes associados a sintomas de ansiedade e depressão em profissionais de saúde que trabalham em Unidades de Terapia Intensiva (UTIs) durante a pandemia de COVID-19 em Bucaramanga e sua área metropolitana.

**Materiais e métodos:**

Este foi um estudo transversal analítico. Ansiedade e depressão foram medidas usando a Escala Hopkins Symptom Checklist-25 (HSCL-25). Análises bivariadas e multivariadas usando regressões lineares foram conduzidas para investigar fatores associados.

**Resultados:**

Um total de 288 pessoas foram incluídas. A prevalência de sintomas de ansiedade e depressão foi de 8,34% (IC 95% 5,41-12,14%). Na análise bivariada, seis fatores foram associados a sintomas de depressão e ansiedade; entretanto, apenas três deles se mantiveram na análise multivariada: ser mulher (β=0,085, IC 95% 0,019 - 0,151), apresentar sintomas de COVID-19 nos últimos 14 dias (β= 0,115, IC 95% 0,024 - 0,205) e ter trabalhado em áreas combinadas de UTI e UTI-COVID-19 (β =0,009, IC 95% 0,025 - 0,173).

**Discussão:**

A prevalência de sintomas de ansiedade e depressão foi consideravelmente menor do que a relatada na literatura científica.

**Conclusões:**

Na população estudada, embora a prevalência de sintomas de depressão e ansiedade tenha sido baixa, foram encontrados três fatores independentes estatisticamente associados à presença desses sintomas mentais.

## Introduction

Pandemics caused by infectious diseases, mainly viral infections, have experienced a resurgence in recent years. Healthcare personnel have faced highly contagious diseases such as SARS, influenza A (H1N1), avian flu, swine flu (H1N1), MERS, and, more recently, COVID-19^1^. The coronavirus disease (COVID-19), caused by the SARS-CoV-2 virus, originated in Wuhan, China, in December 2019 and rapidly spread worldwide, being declared a pandemic by the WHO in March 2020^2^. This viral disease causes severe respiratory problems, requiring hospitalization in many cases, emergency care, or even admission to an Intensive Care Unit (ICU)^3^. In addition to facing the risk of severe illness, healthcare personnel are also at high risk of developing mental health issues, such as anxiety and depression^3^–^5^. 

Multiple factors negatively impact the mental health of ICU healthcare personnel: fear of contagion, long working hours, demanding protocols, uneven patient distribution, feelings of powerlessness, disease complexity, difficult ethical decisions, social isolation, and drastic changes in work methods^4^,^6^,^7^. The consequences of anxiety and depression in this group include reduced quality of life, medical leave, absenteeism, and increased demand for health services^8^. 

Different studies reveal a high prevalence of anxiety and depression among healthcare personnel, especially those caring for COVID-19 patients^8^. It has been observed that one in five healthcare professionals has reported depression and anxiety symptoms, which are more prevalent in women and nursing staff^9^. This has led to the urgent need to implement measures to prevent or reduce these effects^3^. This study is relevant and novel in its analysis of factors associated with anxiety and depression symptoms among ICU healthcare personnel in Bucaramanga, allowing for support strategies tailored to specific local needs. Theoretically, it contributes to understanding how factors such as gender, recent COVID-19 exposure, and work in both general ICUs and COVID-19 ICUs affect mental health. Methodologically, the use of the HSCL-25 ensures the reliability of results. Practically, the findings highlight the need for adapted psychological support policies, improving the healthcare workers' well-being and the responsiveness of the local healthcare system. 

Institutions must implement strategies and spaces to assess the mental health status of healthcare personnel and provide psychological support. The pandemic has highlighted the importance of protecting the well-being of healthcare personnel, who are fundamental pillars in patient care. Therefore, the objective of this study was to evaluate the prevalence and independent risk factors associated with anxiety and depression symptoms among healthcare personnel working in Intensive Care Units (ICUs) during the COVID-19 pandemic in Bucaramanga and its metropolitan area. 

## Materials and Methods

** Design and Population**

An analytical cross-sectional study was conducted on frontline healthcare workers caring for patients diagnosed with COVID-19 in healthcare institutions in Bucaramanga and its metropolitan area between March and April 2021. 

**Eligibility Criteria**

**Figure 1:**
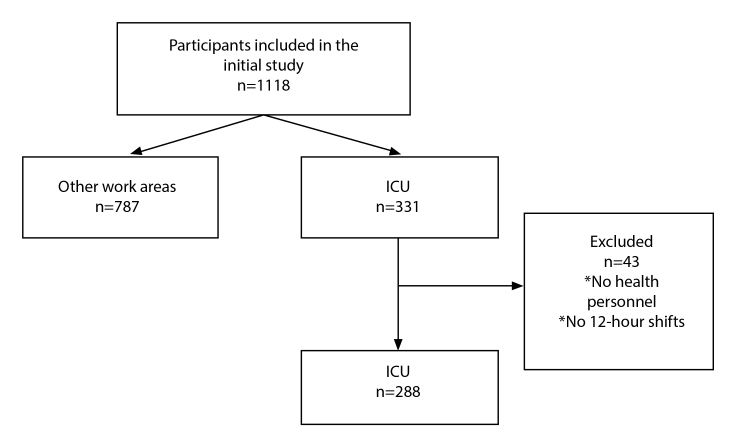
Flowchart of participant selection

This study is a sub-analysis of a previously published research Project, *“Fortalecimiento de Capacidades en Ciencia y Tecnología del Laboratorio de Biología Molecular de la Fundación Cardiovascular de Colombia para atender problemáticas asociadas con agentes biológicos de alto riesgo para la salud humana en Bucaramanga/Santander”*10. For this study, all records of participants from the healthcare area working in the ICUs were included. At the same time, administrative or ancillary staff with shifts of less than 12 consecutive hours were excluded. No sample size calculation was performed; instead, all records of individuals meeting the inclusion criteria and not meeting the exclusion criteria were analyzed, resulting in a total of 288 participants (Figures 1). The initial study sample was selected based on convenience sampling. 

**Measurements**

The dependent variable was the presence of anxiety and depression symptoms, measured using the Hopkins Symptom Checklist-25 (HSCL-25), a shortened version of the Hopkins Symptom Checklist-58 (HSCL-58) was applied. This freely available scale does not require publication permission. It was developed by Derogatis et al.^11^ and later translated and validated into Spanish by Claveria et al.^12^. This checklist consists of two dimensions: the first assesses anxiety symptoms (Items 1-10), while the second focuses on depression symptoms (Items 11-25). Participants respond using a four-point Likert scale ranging from 1 to 4, where 1 represents "Not at all," 2 "A little," 3 "Quite a bit," and 4 "A lot." Originally developed as a self-report symptom inventory, the scale’s total score is obtained by summing item scores and dividing by 25 (the total number of items). A total score ≥ 1.75 raises suspicion for a diagnosis of depression or anxiety. This same cut-off point is applied separately to assess anxiety and depression, using the first 10 and remaining 15 items, respectively. Regarding psychometric properties, the HSCL-25 shows good internal consistency, with a Cronbach's alpha coefficient of 0.92 (α=0.88 for depression and α=0.83 for anxiety). Confirmatory factor analysis revealed the presence of a global factor and two highly correlated factors (0.84). When compared with the gold standard (Composite International Diagnostic Interview-CIDI), the area under the curve (AUC) was 0.89 (95% CI: 0.86-0.93%). For a cut-off point of 1.75, the sensitivity was 88.1% (95% CI: 77.1-95.1%), and the specificity was 76.7% (95% CI: 73.3-79.8%). Furthermore, the scale demonstrated good test-retest reliability, with an intra-class correlation coefficient of 0.92 (95% CI: 0.87-0.95)^13^. 

Sociodemographic characteristics were considered independent variables, which included sex, age, socioeconomic status, municipality of residence, level of education, marital status, household size, mode of transportation to the workplace, and whether any household member had tested positive for COVID-19. Also, personal history was inquired, such as comorbidities, presence of COVID-19 symptoms in the last 14 days, COVID-19 vaccination status, number of vaccine doses received, and presence of COVID-19 antibodies. Finally, work-related aspects were studied, such as the type of healthcare institution, occupation, work area, whether a co-worker had been diagnosed with COVID-19, sharing a break room with co-workers without masks at a distance less than 1 meter for more than 15 minutes, and sharing a dining area with co-workers at a distance less than 1 meter. 

**Information collection procedure**

First, the various healthcare institutions providing services to COVID-19 patients were identified. Subsequently, these institutions were invited to participate in the study. Upon receiving a positive response, they were asked to provide a list of frontline staff or those most exposed to the virus, along with their contact details. The selected personnel were invited to participate in the study by email. In addition, we had the support of the coordination offices of each institution to disseminate the invitation among its staff. The email invitation included a link to an informed consent form and a data collection form developed on the RedCap platform. Once participants completed the form, they obtained a signed copy of the informed consent form and a unique code for identification. 

**Data Set**

The validated information was stored in GitLab^14^. GitLab is an online repository platform based on free software that follows an open-core model, offering a free version that does not require publication permissions. 

**Analysis of data**

A descriptive analysis of the studied population's sociodemographic characteristics and personal and work history was conducted. Categorical variables were presented as absolute and relative values (percentages). In contrast, continuous variables, which did not follow a normal distribution according to the Shapiro-Wilk test, were reported as medians with first and third quartiles (Q1-Q3). Anxiety and depression symptoms were described using absolute and relative values, and the overall prevalence was calculated along with its 95% confidence interval. The dependent variable was treated as a continuous variable; therefore, bivariate and multivariate analyses were conducted using linear regression models. Initially, independent variables were selected based on the background in the literature and biological plausibility for those with an impact on the mental health of healthcare workers in the context of the pandemic. Subsequently, variables with p values ≤ 0.20 were included in multivariate analysis using a backward modeling approach. Finally, variables with p values <0.05 were considered statistically significant, and the assumptions of the linear model were evaluated. All analyses were performed using Stata version 15 statistical software. 

**Ethical Considerations**

The study was conducted following the guidelines of the Declaration of Helsinki and Resolution 8430 of 1993 in Colombia. It was approved by the Research Ethics Committee of the Fundación Cardiovascular de Colombia (protocol code CEI-2020-01858, December 15, 2020). All participants provided written informed consent after being fully informed about the study's objectives, potential risks, and benefits, as well as their right to withdraw at any time without repercussions. To ensure confidentiality, each participant was assigned a unique anonymized code, keeping personal identifiers separate from research data. This information was securely stored in a restricted-access database, accessible only to authorized research team members. 

## Results

**Sociodemographic characteristics, personal and work history**

A total of 288 health personnel participants who worked in the ICUs of eight health institutions in Bucaramanga and its metropolitan area were included in the study. Women accounted for 69.69% of the sample, with a median age of 34 years (Q1=27; Q3=40). It was observed that the majority of participants belonged to a middle socioeconomic status (60.85%) and mainly resided in Floridablanca (43.40%) and Bucaramanga (34.72%). Regarding education, 34.73% had technical training, while 31.60% held university degrees. 

The most common mode of transportation to work was a personal vehicle (33.33%). Regarding personal history, the most frequent comorbidities were obesity (8.33%), COPD or asthma (6.60%), hypothyroidism (4.51%), arterial hypertension (3.13%), diabetes mellitus (2.08%), and dyslipidemia (1.74%). Additionally, 48.06% of participants had completed the vaccination schedule. Regarding job characteristics, 92.36% worked in private healthcare institutions, 37.15% were professional nurses, and 31.38% were nursing assistants. In addition, 21.18% worked in both the general ICU and the COVID-19 ICU. Regarding workplace exposure, 90.97% had a colleague diagnosed with COVID-19, while 83.68% reported not having shared a break room without masks with colleagues (Table 1). 

**Table 1:** Sociodemographic characteristics, personal and work history (n=288)

Characteristics	n (%)
Sociodemographic	
Sex	
Female	200 (69.69)
Male	87 (30.31)
Age (years)*	34 (Q1=27; Q3=40)
Socioeconomic status	
Low	67 (23.84)
Middle	171 (60.85)
High	43 (15.30)
Municipality of residence	
Bucaramanga	100 (34.72)
Metropolitan area**	188 (65.28)
Education level	
Technical training	100 (34.72)
College degree	91 (31.60)
Completed postgraduate education	79 (27.43)
Incomplete postgraduate education	18 (6.25)
Marital status	
Single	139 (48.26)
Married or cohabiting	137 (47.57)
Separated, divorced, or widowed	12 (4.17)
Household size*	3 (Q1=2; Q3=4)
Has anyone in your household been diagnosed with COVID-19? (Yes)	63 (21.88)
Personal history	
COVID-19 symptoms in the past 14 days (Yes)	37 (12.85)
Received COVID-19 vaccine (Yes)	206 (71.53)
COVID-19 vaccine doses received	
First dose	107 (51.94)
Second dose	99 (48.06)
Antibodies against COVID-19	
IgM (+) / IgG (+)	10 (3.47)
IgM (+) / IgG (-)	56 (19.44)
IgM (-) / IgG (+)	44 (15.28)
IgM (-) / IgG (-)	178 (61.81)
Job Characteristics	
Type of healthcare institution	
Private	266 (92.36)
Public	22 (7.64)
Occupation	
Registered nurse	107 (37.15)
Nursing assistant	99 (34.38)
Medical specialist	38 (13.19)
General practitioner	28 (9.72)
Respiratory therapist	8 (2.78)
Physiotherapist	6 (2.08)
Psychologist	2 (0.69)
Work area	
ICU	227 (78.82)
ICU and COVID-19 ICU	61 (21.18)
Has any co-worker been diagnosed with COVID-19?	
Yes	262 (90.97)
No/don’t know	26 (9.03)
Have you shared a break room with co-workers without masks and at a distance of less than 1 meter for more than 15 minutes? (Yes)	47 (16.32)
Have you shared a dining area with co-workers at a distance of less than 1 meter? (Yes)	99 (34.38)

*Median (quartile 1 (Q1) and quartile 3 [Q3]). ** Floridablanca, Piedecuesta, Girón, and Lebrija

**Prevalence of anxiety and depression**

The combined prevalence of anxiety and depression symptoms was 8.34 % (95% CI: 3.41-12.14%).

**Independent risk factors associated with anxiety and depression symptoms**

In the bivariate analysis, six independent variables were associated with anxiety and depression: sex, as women had a higher prevalence compared to men (p=0.025); household size (p=0.046); experiencing COVID-19 symptoms in the past 14 days (p=0.012); not being vaccinated against COVID-19 (p=0.033); occupation, as registered nurses had a higher prevalence compared to medical specialists (p=0.030), and working in the ICU or COVID-19 ICU (p=0.017) (Table 2).

**Table 2:** Bivariate analysis of independent risk factors associated with anxiety and depression symptoms (n=288)

Characteristics	Coefficient (β)	CI 95%	p-value*
Sex			
Male	Reference		
Female	0.076	0.009 - 0.143	0.025
Age (years)*	-0.002	-0.005 - 0.002	0.333
Socioeconomic status			
Low	Reference		
Middle	-0.009	-0.083 - 0.063	0.788
High	-0.023	-0.122 - 0.076	0.650
Education level			
Incomplete/completed postgraduate	Reference		
Technical training	-0.006	-0.080 - 0.068	0.882
Academic	0.027	-0.049 - 0.103	0.489
Marital status			
Single	Reference		
Married or cohabiting	-0.033	-0.096 - 0.030	0.307
Separated. divorced or widowed	-0.080	-0.237 - 0.077	0.316
Household size	0.019	0.0003 - 0.037	0.046
Has anyone in your household been diagnosed with COVID-19?			
No	Reference		
Yes	0.027	-0.047 - 0.102	0.475
Presence of comorbidities			
No	Reference		
Yes	0.069	-0.004 - 0.143	0.064
COVID-19 symptoms in the past 14 days			
No	Reference		
Yes	0.117	0.026 - 0.208	0.012
Vaccine against COVID-19			
Yes	Reference		
No	0.074	0.006 - 0.141	0.033
Type of healthcare institution			
Private	Reference		
Public	-0.021	-0.137 - 0.095	0.720
Occupation			
Medical specialist	Reference		
Nursing assistant	0.044	-0.056 - 0.143	0.387
Registered nurse	0.109	0.010 - 0.207	0.030
General practitioner	0.097	-0.032 - 0.227	0.141
Respiratory therapist	0.122	-0.080 - 0.325	0.235
Physiotherapist and psychologist	0.017	-0.185 - 0.219	0.866
Work area			
ICU	Reference		
ICU and COVID-19 ICU	0.091	0.016 - 0.166	0.017
Has any co-worker been diagnosed with COVID-19?			
No/don’t know	Reference		
Yes	0.032	-0.075 - 0.139	0.559
Have you shared a break room with co-workers without masks and at a distance of less than 1 meter for more than 15 minutes?			
No	Reference		
Yes	0.081	-0.002 - 0.164	0.055
Have you shared a dining area with co-workers at a distance of less than 1 meter?			
No	Reference		
Yes	0.057	-0.007 - 0.122	0.082

* p-value from a linear regression; CI: confidence interval.

Finally, in the multivariate analysis, three variables were associated with anxiety and depression symptoms: female sex (female vs. male; β=0.085; 95% CI: 0.019 - 0.151), presence of COVID-19 symptoms in the past 14 days (Yes vs. No; β=0.115; 95% CI: 0.024 - 0.205), and work area (ICU vs. ICU and COVID-19 ICU; β=0.099; 95% CI: 0.025 - 0.173) (Table 3). These variables explained 5.27% of the variability in anxiety and depression symptoms (adjusted R-squared). The model did not meet the assumptions of normality or homoscedasticity, but no collinearity was evident (variance inflation factor < 10 for all variables). 

**Table 3:** Multivariate analysis of risk factors associated with anxiety and depression symptoms

Characteristics	Coefficient (β)	CI 95%	p-value*
Sex			
Male	Reference		
Female	0.085	0.019 - 0.151	0.011
COVID-19 symptoms in the past 14 days			
No	Reference		
Yes	0.115	0.024 - 0.205	0.014
Work area			
ICU	Reference		
ICU and COVID-19 ICU	0.099	0.025 - 0.173	0.009

*p value from a linear regression; CI: confidence interval.

## Discussion

In this cross-sectional study of ICU healthcare professionals during the COVID-19 pandemic, a low prevalence of anxiety and depression symptoms was observed. Three factors were associated with these symptoms: Female sex, presence of COVID-19 symptoms in the past 14 days, and work area. 

The prevalence of anxiety and depression symptoms in this study was considerably lower than that reported in other studies. A systematic review^9^ evaluating 24 studies showed a prevalence of anxiety from 14.5% to 44.6% and depression from 8.9% to 50.4%. Another systematic review and meta-analysis conducted by Salari et al.^8^ also presented prevalence rates similar to those previously reported, with a prevalence of depression of 24.3% and a prevalence of anxiety of 25.8%. Other cross-sectional studies, such as that by Peng et al.^15^, showed that up to 65.9% and 58.7% of ICU workers presented symptoms of depression and anxiety, respectively, with no significant difference between first-line and second-line workers in contact with COVID-19 patients. The lower prevalence we observed may be due to sample size, the instruments used to measure anxiety and depression, the sociodemographic characteristics of the study population, and the timing of the research (second wave of the COVID-19 pandemic). 

Regarding the factors associated with the development of anxiety and depression symptoms, our study found that being a woman was associated with a higher prevalence rate, similar to what has been reported in the literature^9^,^15^. This association is likely since the nursing staff, where women constitute the majority, had the greatest exposure to COVID-19-infected patients^16^. On the other hand, our study showed a significant relationship between experiencing COVID-19 symptoms in the past 14 days and the presence of anxiety and depression symptoms. This association was also observed in other studies. Motahedi et al.^17^ found higher anxiety levels among healthcare workers with prior COVID-19 infection. Chen et al.^18^ showed the presence of respiratory symptoms as an independent risk factor for suffering from anxiety and depression. The above findings could be influenced by health professionals' fear of transmitting the virus to their family members^9^. 

The work area was also identified as an associated risk factor, with a higher prevalence of mental symptoms among participants who worked shifts in both the ICU and the COVID-19 ICU. This association was predictable, given the high workload experienced by these health professionals, as demonstrated by Peng et al.^15^, who found that longer working hours in the ICU were related to a deterioration in mental health. This situation was significantly exacerbated in referral hospitals, where patient volume was higher^19^. This study included institutions that faced this reality while meeting local and national healthcare demands. 

Among the strengths of this study, we highlight that it included and evaluated a sample of health workers from various institutions, especially those at higher risk of presenting any mental health symptoms related to the COVID-19 pandemic, such as ICU staff, without making distinctions based on healthcare professions. However, a significant limitation is that the sample size was selected in a non-probabilistic and limited way, which could introduce selection bias and limit the ability to find associations with some factors due to insufficient statistical power. Therefore, the results of the study are not generalizable to the entire population of healthcare workers. Additionally, the model had limited explanatory power of the outcome of interest, and some assumptions were not met. This may be explained by the fact that it was a secondary data analysis, and it is possible that relevant variables were not included in the initial study; however, it is worth noting that the associated factors found have biological plausibility and are supported by findings from previous research. 

## Conclusions

This study identifies female sex, experiencing COVID-19 symptoms in the past 14 days, and working in specific work areas as factors associated with the risk of presenting anxiety and depression symptoms. However, in our study population, the prevalence of these symptoms was low. The higher prevalence of mental health symptoms among nursing professionals is notable and underlines the need to pay them special attention, especially to their workload. Further long-term research is needed to evaluate the impact of the COVID-19 pandemic on the mental health of healthcare workers. 
